# Cue-Reactivity Among Young Adults With Problematic Instagram Use in Response to Instagram-Themed Risky Behavior Cues: A Pilot fMRI Study

**DOI:** 10.3389/fpsyg.2020.556060

**Published:** 2020-11-02

**Authors:** Nisha Syed Nasser, Hamed Sharifat, Aida Abdul Rashid, Suzana Ab Hamid, Ezamin Abdul Rahim, Jia Ling Loh, Siew Mooi Ching, Fan Kee Hoo, Siti Irma Fadillah Ismail, Rohit Tyagi, Mazlyfarina Mohammad, Subapriya Suppiah

**Affiliations:** ^1^Department of Radiology, Faculty of Medicine and Health Sciences, Universiti Putra Malaysia, Serdang, Malaysia; ^2^Department of Imaging, Faculty of Medicine and Health Sciences, Universiti Putra Malaysia, Serdang, Malaysia; ^3^Department of Family Medicine, Faculty of Medicine and Health Sciences, Universiti Putra Malaysia, Serdang, Malaysia; ^4^Neurology Unit, Department of Medicine, Faculty of Medicine and Health Sciences, Universiti Putra Malaysia, Serdang, Malaysia; ^5^Department of Psychiatry, Faculty of Medicine and Health Sciences, Universiti Putra Malaysia, Serdang, Malaysia; ^6^Aerobe Pte Ltd., Singapore, Singapore; ^7^Center for Diagnostic, Therapeutic and Investigative Studies, Faculty of Health Sciences, Universiti Kebangsaan Malaysia, Bangi, Malaysia

**Keywords:** internet addiction, behavioral addiction, emotional cues, BOLD signal, response to reward, social networking

## Abstract

**Background:**

Problematic Instagram use (PIGU), a specific type of internet addiction, is prevalent among adolescents and young adults. In certain instances, Instagram acts as a platform for exhibiting photos of risk-taking behavior that the subjects with PIGU upload to gain likes as a surrogate for gaining peer acceptance and popularity.

**Aims:**

The primary objective was to evaluate whether addiction-specific cues compared with neutral cues, i.e., negative emotional valence cues vs. positive emotional valence cues, would elicit activation of the dopaminergic reward network (i.e., precuneus, nucleus accumbens, and amygdala) and consecutive deactivation of the executive control network [i.e., medial prefrontal cortex (mPFC) and dorsolateral prefrontal cortex (dlPFC)], in the PIGU subjects.

**Method:**

An fMRI cue-induced reactivity study was performed using negative emotional valence, positive emotional valence, and truly neutral cues, using Instagram themes. Thirty subjects were divided into PIGU and healthy control (HC) groups, based on a set of diagnostic criteria using behavioral tests, including the Modified Instagram Addiction Test (IGAT), to assess the severity of PIGU. In-scanner recordings of the subjects’ responses to the images and regional activity of the neural addiction pathways were recorded.

**Results:**

Negative emotional valence > positive emotional valence cues elicited increased activations in the precuneus in the PIGU group. A negative and moderate correlation was observed between PSC at the right mPFC with the IGAT scores of the PIGU subjects when corrected for multiple comparisons [*r* = −0.777, (*p* < 0.004, two-tailed)].

**Conclusion:**

Addiction-specific Instagram-themed cues identify the neurobiological underpinnings of Instagram addiction. Activations of the dopaminergic reward system and deactivation of the executive control network indicate converging neuropathological pathways between Instagram addiction and other types of addictions.

## Introduction

In the past decade, social media addiction has become prevalent among adolescents and young adults worldwide (Franchina et al., 2018; Kircaburun and Griffiths, 2018). There are many internet-based social networking applications (SNAs) that can lead to social media addiction, namely Facebook, WeChat, and Instagram. Instagram is an SNA that uses images as a means of communication, providing a platform for photo-sharing and displaying advertisements in the form of photographs, short videos, carousels, and stories (Huang and Su, 2018). Instagram was launched in 2010, and within 8 years it has surpassed Facebook users by garnering 700 million monthly active users; the majority being young adults aged between 18 and 29 years old (Pew Research Center, 2018).

While Instagram can provide a fun means for social networking, there is a dark side to it. A systematic review of nine studies, involving 510 participants, highlighted that the problematic use of SNAs is associated with psychiatric disorders and risk-taking behaviors (Hussain and Griffiths, 2018). Many individuals have reportedly died while attempting to take a selfie in 52 incidents worldwide, with the mean age of the victims being 23.3 years (Hussain and Griffiths, 2018). Hence, social media addiction is a specific type of internet addiction that can result in negative social, medical, and psychological consequences (Brand et al., 2016).

The motivation to use an SNA is related to the need for social communication and peer acceptance, which is influenced by the users’ personalities (Seidman, 2013; Kuss and Griffiths, 2017). The use of SNAs have become more rampant with the wide availability of smartphones while the fear of not having a mobile phone has been termed nomophobia. Nomophobia potentially causes susceptible users to use the smartphones impulsively, and this increases their vulnerability to develop addiction-related symptoms (Kuss and Griffiths, 2017). Social media addiction is believed to share converging neurobiological pathways with substance use disorders (SUDs) and other behavioral addictions such as online pathological gambling (PG), pathological gaming, pornography, and problematic e-shopping (Brand et al., 2016). Several studies have attempted to explore the neurobiological underpinnings of social media addiction pertaining to various social media platforms, namely Facebook, WhatsApp, and WeChat (Turel et al., 2014; Wegmann et al., 2015; Leng et al., 2019).

These studies aim to present the addicted individuals with stimuli that mimic the online environment. The addicted individuals perceived the stimuli as a rewarding experience, and achieved a sense of gratification that interacts with different cognitive and affective neuronal processes (e.g., cravings, cue-reactivity, and mood management) (Wegmann and Brand, 2019). Furthermore, the perceived rewarding experience by young adults is evident from the type of pictures with catchy hashtags (“#”) that they upload to Instagram. These photos tend to generate the highest number of likes, i.e., PIGUs gain attention and popularity among their peers (Kurniawan et al., 2017). Data mining of publicly available Instagram uploads has revealed a trend toward receiving likes for images known as negative emotional valence type of pictures. Selfies related to illicit drugs use and alcoholism, as well as selfies taken from heights and dangerous places, selfies taken while driving, and selfies taken while performing dangerous stunts; have been increasingly uploaded by users particularly in the young adult age group (Kurniawan et al., 2017; Zhou et al., 2017; Sherman et al., 2018b). Hence, Instagram addiction, concerning the attempts to get more online likes, has been attributed to the desire to gratify the need for peer acceptance and their perceived reward of gaining social popularity (Kircaburun and Griffiths, 2018).

Neuroimaging studies, which include resting-state and task-based fMRI, investigate *in vivo* interactions that occur in the neural substrates, thus enabling the assessment of the response to reward in internet addictions (Sharifat et al., 2018). Primarily, fMRI studies have identified the involvement of the mesocorticolimbic dopaminergic system, also called the dopaminergic reward network, which acts as the basis of explaining the brain’s response in behavioral addictions (Wang et al., 2016; Syed Nasser et al., 2019a). The activation of the dopaminergic reward network, consisting of the amygdala-striatal system, i.e., the amygdala (Amyg), ventral striatum (VS), caudate nuclei, hippocampus (HIPP), insula, nucleus accumbens (NAcc), and precuneus (Prec) propels the addicted individuals to approach and persistently seek the addictive stimulus (Pierce and Kumaresan, 2006; Gardner, 2011; Turel et al., 2014; Wegmann et al., 2015; Sherman et al., 2016; Starcke et al., 2018; Leng et al., 2019). Cue-induced reactivity paradigms and structural MRI studies have also been utilized to evaluate the neurobiology of Facebook and WeChat usage among the youth (Sherman et al., 2016, 2018a; Turel and Bechara, 2016; He et al., 2017; Wegmann et al., 2018; Leng et al., 2019). The activation of the dopaminergic reward network, in particular the NAcc, Prec, and Amyg, was observed when the addicted individuals were presented with addiction-specific stimuli (Ko et al., 2009; Turel et al., 2014; Sherman et al., 2018a; Wegmann and Brand, 2019). Thus, we formulate our first hypothesis:

Hypothesis 1: Subjects with PIGU will exhibit increased activation of the dopaminergic reward system compared to healthy controls when presented with Instagram-themed cues.

Conversely, the executive control network (ECN) has also been implicated in the response to reward in addiction. The ECN involves the prefrontal cortex (PFC), predominantly the medial prefrontal cortex (mPFC) and the dorsolateral prefrontal cortex (dlPFC), with the former demonstrating robust activity and the latter revealing deactivations during response to reward tasks (Goldstein and Volkow, 2011). The activation of the dorsal mPFC increased during tasks that required internally directed cognition, i.e., assigning incentive salience in the case of addiction whereas deactivation in the ventral mPFC was noted consistent with a competitive relationship between cognitive and emotional processing that might be subserved by these separate regions (Zhang and Volkow, 2019). Moreover, the PFC, inclusive of the ventral and dlPFC, is implicated in the automatic response tendencies to approach or avoid a stimulus. Specifically, the dlPFC is activated to bring into effect one’s approach toward a positive emotional valence stimulus (congruent affective response); but is deactivated when an addicted individual approaches a negative emotional valence affective stimulus (incongruent affective response) (Goldstein and Volkow, 2011; Volman et al., 2011).

Furthermore, Wei et al. (2017) summarized the interactions of the neural substrates succinctly in their tripartite neurocognitive model of internet gaming addiction that proposed interconnectivity of the nodes in the hyperactive-impulsive system with the hypoactive reflective ECN (Wei et al., 2017). Specifically, dysregulation of the ECN is postulated to occur, whereby in general, it fails to exert an inhibitory control in addicts (Montag et al., 2018; Wegmann et al., 2018). Hence, we postulate our second hypothesis:

Hypothesis 2: Content-specific Instagram-themed cues with a high number of likes compared to neutral cues will cause the deactivation of the executive control network system among subjects with PIGU.

Thus, we designed a validated cue-reactivity fMRI paradigm that utilized addiction-specific Instagram-themed cues and contrasted it with neutral cues (Syed Nasser et al., 2020b). The concept of the truly neutral cues or pictures was adapted from the International Affective Picture System (Lang et al., 2008) which depicts pictures of the environment and inanimate objects with either a grayscale background or non-vivid colors in the background that are unlikely to induce any strong emotional response. Whereas the negative emotional valence cues were tailored to depict risky Instagram posts that had a high number of likes.

The objectives of our study were, firstly, to identify whether addiction specific cues can cause the activation of the dopaminergic reward system in problematic Instagram users and secondly, to identify whether the same cues can cause the deactivation of the ECN. We also aimed to correlate the intensity of the neural responses, with regards to nodes in the reward system and ECN system in response to the negative emotional valence images, with the severity of PIGU.

To the best of our knowledge, this is the first cue-reactivity study that has utilized fMRI to assess the neural mechanisms of Instagram-based social media addiction and to have comparisons made with control subjects.

## Materials and Methods

Institutional ethical approval (UPM/TNCPI/RMC/1.4.18.2/JKEUPM) was received to conduct this study. The sample population was recruited in two phases.

### Participants

The first phase of this study utilized several online questionnaires that surveyed the demographic data and behavioral data of the subjects. The participants were undergraduate university students of Asian descent at a local public university. All the subjects owned a smartphone and were active Instagram users. The exclusion criteria were respondents who had any substance dependence, including to cigarettes or alcohol, and those who had a history of consumption of psychotropic drugs or long-term medications. Respondents who had a history of head trauma were also excluded from the study. We also excluded respondents who wore spectacles due to visual acuity problems, and those who had any self-reported underlying psychiatric disorders, e.g., obsessive-compulsive disorder, and major depression. We also excluded all respondents through our psychological assessment and who had high DASS-21 scores, as it indicated the possibility of major depression, severe anxiety, or extreme stress.

Subjects were invited to participate in the survey via advertisements made on student bulletin boards. The students were not given any rewards for participating in this study, and potential participants were also informed that their acceptance or refusal to participate in this study would not affect their academic grades. The questionnaires that assessed their behavioral data included Barrett’s impulsivity test (BIS-11), and the Depression, Anxiety, and Stress Scores (DASS-21) assessment test (Parkitny and McAuley, 2010; Syed Nasser et al., 2019b), the results of which are published in a separate publication (Syed Nasser et al., 2020a). The Smartphone Addiction Scale-Malay version (SAS-M) questionnaire (Kwon et al., 2013; Ching et al., 2015) and the Modified short Instagram Addiction Test (IGAT) Kircaburun and Griffiths (2018) questionnaire were also utilized.

All respondents were informed about the study procedures and gave written informed consent according to the Declaration of Helsinki. A total of 1,060 respondents returned completed survey forms. Based on the scores achieved, we performed simple random sampling and contacted a representative group of respondents via telephone. The principal investigator invited several eligible respondents to participate in the second phase of this study that involved a cue-reactivity task-based fMRI study. The fMRI subjects were categorized into PIGU and healthy control (HC) groups based on their overall performance in the previously administered behavioral tests. The inclusion criteria for our fMRI study subjects were that they should be citizens of Asian descent, right-handed, have good visual acuity, and fulfilled the required scores based on the behavioral test questionnaires. The inclusion criteria for HC were that their performance on the modified diagnostic criteria and questionnaire scores should be below the range of the PIGU subjects and they did not experience any deterioration in studies and interpersonal relationship due to their use of Instagram. Exclusion criteria included factors that would cause the potential subjects to be contraindicated for an MRI scan.

### Behavioral Test Questionnaires

#### Smartphone Addiction Scale – Malay Version

We used a modified version of the diagnostic criteria for smartphone addiction by Lin et al. (2016) (see Supplementary File 1) in combination with the Smartphone Addiction Scale- Malay version (SAS-M) questionnaire (see Supplementary File 2). The SAS-M is a validated 33-point questionnaire which used a 6-point Likert scale, ranging from 1 to 6 (1 = ‘strongly disagree’ to 6 = ‘strongly agree’) to assess for typical smartphone addiction symptoms such as salience, withdrawal, loss of control, relapse, and conflict. These were based on the domains for a cyberspace-orientation relationship, daily life disturbance, primacy, overuse, positive anticipation, and withdrawal. The questionnaire was interpreted based on a cut-off score of ≥98 that gave a sensitivity of 71.43%, a specificity of 71.03%, PPV of 64.10%, and NPV of 77.44% for detecting problematic smartphone use (Ching et al., 2015). Given its validity and reliability (Cronbach’s alpha = 0.94), the total score of all items was calculated, which represented the severity of the addiction symptoms of each subject.

### Modified Instagram Addiction Test (IGAT) Questionnaire

We also used the modified short Young’s Internet Addiction Test (s-IAT), a 12-item questionnaire with a Likert scale of 1 to 5, to assess for internet addiction, developed by Pawlikowski et al. (2013). We adapted it to develop our modified Instagram Addiction Test (IGAT) questionnaire (see Supplementary File 3), which is similar to the method employed by Khazaal et al. (2015) for online gamers and Kircaburun and Griffiths (2018) for Instagram users (Khazaal et al., 2015; Kircaburun and Griffiths, 2018). The test included factors that were crucial to the development of Instagram addiction namely loss of control and time management as well as the development of ‘cravings’ and social problems related to its pathological usage (Pawlikowski et al., 2013). We developed the IGAT questionnaire from the s-IAT by changing the words ‘online’ to ‘Instagram.’ In combination with the other criteria, we used a cut-off score of ≥37 for our IGAT as an indicator of PIGU. We defined the cut-off score using data from a previous study by Meerkerk (2007), which advocated that the behavior or symptoms specified in a questionnaire assessing addictive or compulsive internet use should be rated on average as ‘sometimes’ in all items. A cut-off score of 36 was achieved when selecting the average score of ‘3’ for all 12 items in the questionnaire. For defining the cut-off score based on the data, specific predictive values of the two s-IAT factors (‘loss of control’ and ‘craving’) with respect to the validation constructs were calculated. Based on these data, the cut-off score indicating pathological use (*M* + 2 *SD*) would be >37. This score is quite comparable to the cut-off score of 36, following the plausible content-based argument by Meerkerk (2007).

### Protocol for fMRI Experiment

#### In Scanner Cue-Reactivity Paradigm

The fMRI scans were performed within the 2 weeks after the completion of the behavioral test questionnaires. A total of 36 subjects were eligible for the second phase of this study and were recruited for fMRI scans. The subjects were positioned supine on the fMRI scanner bed to view the task back-projected onto a screen through a mirror attached to the head coil. Head motion was minimized by using foam pads. The stimuli were presented, and the timing of all stimuli and responses were achieved using Matlab (Mathworks) and a block design fMRI software, Nordic Aktiva (Aerobe Pte. Ltd., Singapore). The participants’ responses were collected online using an MRI-compatible button box. Before conducting the scan, the subjects underwent a routine physical examination (height, weight, and blood pressure measurements) and completed the MRI safety checklist.

We designed and validated a novel Go/No-go type of cue-reactivity paradigm that simulated the online environment of Instagram (Syed Nasser et al., 2020b,c). We used experimental conditions that comprised of a set of randomly displayed validated picture flashcards. An out-of-scanner validation of the pictures was performed using an impartial subset of the respondents from phase 1 of this study. The response of the small subset of participants (*N* = 40) objectively scored a database of 200 pictures based on the motivation, emotional valence, and arousal induced by the corresponding pictures. An optimized selection of pictures was made to create a validated database of pictures with Instagram-themed cues (Syed Nasser et al., 2020b,c).

We assessed the in-scanner detected neural responses of the PIGU and HC subjects toward the Instagram-themed pictures. The first condition termed as the negative emotional valence/risky with a high number of likes cues, was designed to elicit ‘cravings’ among the PIGU group, and depicted risqué selfies taken from heights, on railway tracts, during texting and driving, and while performing dangerous stunts (Syed Nasser et al., 2020b). The second condition was positive emotional valence Instagram cues that had an assorted number of high and low likes, which comprised of colorful, pleasant pictures taken from the subjects’ own Instagram account and did not contain any risqué behavior. These pictures were considered socially acceptable and pleasant pictures, designed not to elicit any marked ‘cravings’ in both the PIGU and HC subjects. Lastly, truly neutral cues with a low number of likes, which depicted pictures of inanimate objects and landscapes taken in grayscale, acted as a baseline control condition for both groups (Syed Nasser et al., 2020b).

The experimental paradigm, having three conditions, was presented in a block design that contained eight blocks per condition. The three conditions were presented alternatively, each having five cues per block. Each cue was shown for 6 s with a block lasting for 30 s. After each block, a fixation on a black background was displayed for 30 s. The overall presentation of 24 blocks, followed by immediate fixation lasted for 24 min in total (see Supplementary Figure 1).

The experimental cues displayed several things on its interface, including (i) two buttons; “Like” or “Pass,” (ii) Instagram logo menu bar, (iii) the total number of likes received for the image, and (iv) the hashtag (#) that accompanied the image. The cues were randomly assigned a “popular” value of 23–45 likes or an “unpopular” value of 0–22 likes as described by Sherman et al. (2018a). The participants indicated their response on whether they felt ‘arousal’ or ‘cravings’ for Instagram when viewing the pictures. This was done by performing a binary decision-making task of selecting a button from two options available on an MRI-compatible response box. They conveyed their selection by using their right thumb to indicate “Like” and their left thumb to indicate a “Pass” response toward the projected pictures, respectively.

The handling of the motor action’s neural activity mapping had to be counterbalanced and negligible, as this was controlled for in the threshold setting and masking of conditions. Furthermore, block-designed fMRI presupposes the steady-state of regional cerebral blood flow and has been applied to examinations of brain activations caused by tasks requiring sustained or repetitive movements (Toma and Nakai, 2002). Hence, any activations in the supplementary motor area will not exhibit any significant percentage signal change (PSC) in the ROI analysis, as is the case with the activations in the visual cortex.

### MRI Data Acquisition and Analysis

Structural images were acquired using a 3.0 Tesla MRI Scanner (Siemens MAGNETOM Prisma, Siemens Medical Solutions, Erlangen, Germany). The participants from the two groups underwent a T1-weighted magnetization-prepared rapid gradient echo (MPRAGE) structural brain imaging [TR = 2,300 ms, TE = 2.27 ms, FOV = 250 mm, slice thickness = 1 mm, number of slices per slab (sagittal) = 160, voxel size = 1.0^∗^1.0^∗^1.0 mm]. Structural imaging was followed by the cue-reactivity task, which was performed for 24 min using the BOLD imaging sequence.

BOLD, echoplanar imaging (EPI) sequence was performed (TR = 3,000 ms, TE = 30.0 ms, FOV = 220 mm and voxel size = 2.3^∗^2.3^∗^3.0 mm), having 34 contiguous axial slices at 3 mm thickness covering the entire brain, in a phase-encoding direction from an anterior to posterior direction. The stimuli, in the form of picture flashcards, were transmitted from the Nordic Aktiva software to an MRI compatible computer screen. The participants viewed the images for the stipulated duration of time and recorded their responses toward the paradigm. During image acquisition, the timing of the picture projections were synchronized with the timing of the BOLD signal detection. The objective emotional responses of the subjects were recorded simultaneously.

### fMRI Data Analysis

fMRI data analysis was carried out using the neuroimaging software, Statistical Parametric Mapping (SPM 12)^1^ and all data were pre-processed for slice timing corrections, realigned for motion corrections, normalized to the standard Montreal Neurological Institute (MNI) template, and smoothed with a Gaussian filter of 6 mm FWHM.

Subsequently, 1st level analysis was carried out using a general linear model (GLM) for the determination of the brain voxels that were activated during each type of condition. At the subject level, the onset timings for all block conditions, i.e., negative emotional valence, truly neutral and positive emotional valence Instagram cues were entered to the design matrix for both the PIGU and HC groups. Next, negative emotional valence vs. truly neutral, positive emotional valence vs. truly neutral and negative emotional valence vs. positive emotional valence contrasts were calculated.

During the 2nd level analysis, a 2 × 3 flexible factorial model was performed at the group level, involving the two groups (PIGU and HC groups) and the three main conditions, using inter-subject variability as a random effect analysis. The voxels that significantly differed in the BOLD signal during negative emotional valence, truly neutral and positive emotional valence conditions between the PIGU and control group (PIGU > control group) were evaluated. Results were then family-wise error (FWE) corrected using a cluster-based threshold of 0.05. Following this, the BOLD signal from the peak voxel within each cluster that demonstrated between-group differences was extracted using the Wake Forest University PickAtlas (WFU) software^2^.

### ROI Analysis

Based on *a priori* knowledge, we selected 6 ROIs specifically involved in the dopaminergic reward network and the executive control network seeds, i.e., including the Amyg, ACC, Prec, OFC, dlPFC, and mPFC (Everitt and Robbins, 2005; Pierce and Kumaresan, 2006; Ko et al., 2009; Haber and Knutson, 2010; Liu et al., 2011; Starcke et al., 2018; Wegmann et al., 2018). The anatomical ROIs were created using the automated anatomical labeling atlas (AAL) template provided by the WFU PickAtlas toolbox. The PSC for each ROI was calculated by averaging the signals across all the voxels within the specific ROI using the MarsBaR SPM toolbox^3^. A Mann–Whitney *U* test was performed to assess for any significant difference in the PSC between groups. Bonferroni correction for multiple comparisons was performed for significant levels. Finally, correlation analyses of PSC at the selected ROIs in the negative emotional valence condition with the IGAT scores were performed using IBM SPSS 22 (IBM Released 2013, Armonk, NY, United States: IBM). Owing to the discrete thresholding of IGAT scores and the skewness of distributions of the other measures, Spearman’s Rho was used to compute covariance. Bonferroni correction for multiple comparisons was used for *post hoc* comparisons.

## Results

### Demographics of the Participants

From the first 36 subjects recruited for the fMRI study, six were removed from the final analysis as their fMRI results had a weak signal to noise ratio (SNR) caused by movement artifacts. Thus, there were a total of 30 subjects in the final fMRI analysis (15 PIGU subjects and 15 HC subjects). Table 1 shows the demographic data of age, SAS-M score, and modified IGAT score, for the PIGU group and HC group. The control group consisted of 53% females (*n* = 8) and 47% males (*n* = 7). In the PIGU group, the majority were males (*n* = 10; 67%) followed by 33% females (*n* = 5) with the mean age of 22.2 ± 0.86 years, having a mean duration of smartphone use of 6.8 years, with the daily frequency of using the smartphone for 7.47 h ± 4.47, and 4.33 h ± 1.35 average daily hours of being active on Instagram (Table 1). The scores attributed by the participants for various dimensions of the SAS-M questionnaires are shown in Supplementary Table 1.

**TABLE 1:** Demographic data of psychosocial factors, smartphone, and Instagram usage patterns between the PIGU group and control group.

	PIGU group		Control group		
	Mean ± *SD*	Minimum–Maximum	Mean ± *SD*	Minimum–Maximum	*p*-value
Subject’s age (years)	22.2 ± 0.86	21–24	21.67 ± 1.18	20–23	0.168
Duration of SPU (years)	6.8 ± 1.82	4–10	5.03 ± 1.85	3–9	0.014*
Avg. SPU time (hours)	7.47 ± 4.47	2–17	3.67 ± 2.32	0.5–8	0.007*
Avg. Instagram use time (hours/day)	2.46 ± 1.40	1–6	0.88 ± 0.45	0–1.5	0.001*
Duration on Instagram (years)	4.33 ± 1.35	2–6	3 ± 1.93	0–7	0.036*
Texting (hours/day)	3.93 ± 0.79	3–5	3 ± 1.30	1–5	0.026*
Updating stories (hours/day)	3.4 ± 1.05	2–5	2.4 ± 0.91	1–4	0.010*
Live video streaming (hours/day)	1.86 ± 0.91	1–4	1.4 ± 0.73	1–3	0.135
Video call (hours/day)	2.33 ± 0.97	1–4	1.66 ± 0.97	1–4	0.072
Depression	11.07 ± 7.32	2–22	5.07 ± 5.18	0–16	0.016*
Anxiety	10.40 ± 6.24	2–24	4.93 ± 2.60	0–10	0.009*
Stress	13.73 ± 7.70	2–24	5.60 ± 5.41	0–18	0.003*
BIS	73.07 ± 66.8	62–85	66.8 ± 5.77	55–76	0.010*

*Significant at p < 0.05. SPU, smartphone usage; PIGU, problematic Instagram usage; Avg, average; BIS, Barrett’s impulsiveness scale.

### Task-Based fMRI Results

The response time that was taken by the PIGU when viewing the negative emotional valence images before indicating their preferred response, i.e., to “Like” or to “Pass” was longer than the time taken by the control group. The average number of times the participants responded to “Like” the risky images was significantly higher in the PIGU compared to the control group (see Table 2 and Supplementary Table 2).

**TABLE 2:** Response time and response type based on cue-reactivity toward different conditions among the PIGU and healthy control groups.

	Response time (ms)			Number of response type (choose ‘Like’ or choose ‘Pass’)			
	Positive emotional valence images	Negative emotional valence images	Neutral images	Negative emotional valence press like	Negative emotional valence press pass	Neutral press like	Neutral press pass
**PIGU**							
Mean	69.977	82.475	75.218	17.307	24.2	21.6	20.307
*SD*	27.792	30.944	25.585	7.909	8.334	8.304	7.134
**HC**							
Mean	56.266	69.560	66.025	10.133	29.866	18.142	22.666
SD	8.072	14.341	13.424	10.07	10.07	8.899	9.131
p-value	0.085	0.158	0.228	0.048*	0.104	0.289	0.458

*Significant at p < 0.05.

Anatomical localization of the significant clusters for 2^∗^3 factorial analysis is reported in Table 3. There was no significant group ^∗^ condition interaction at the whole-brain level; pFWE < 0.05. The between-group whole-brain activation in the PIGU group compared to the control group condition (PIGU group > Control group) and control group compared to the PIGU group condition (Control group > PIGU group) while viewing truly neutral pictures are shown in Supplementary Tables 3, 4. All these regions showed significant activation at pFWE < 0.05. When processing truly neutral cues, the PIGU group as compared to the control group (PIGU group > control group) exhibited increased activation, predominantly in the occipital and frontal lobes. Subjects in the control group (Control group > PIGU group) demonstrated increased activation of the calcarine cortex and the right MOG when viewing truly neutral cues.

**TABLE 3:** Anatomical localization of the significant clusters for 2*3 factorial analysis; *p* < 0.05, FWE corrected.

Anatomical localization of cluster	Coordinates of global maxima			Mean *T*	Peak *T*	Voxels
**Main effect of group PIGU > HC (*k* > 20)**						
L fusiform	−46	−62	−14	6.95	12.58	132
R lingual gyrus	22	−86	8	7.09	12.32	241
L middle frontal gyrus	−38	2	22	6.58	11.11	678
L middle occipital gyrus	−20	−92	6	6.94	10.96	215
R superior parietal gyrus	22	−64	52	6.54	10.44	194
R inferior frontal gyrus	40	12	30	6.38	9.45	130
L superior parietal gyrus	−30	−74	48	6.22	8.90	71
L inferior parietal gyrus	−32	−50	56	6.05	8.84	260
R inferior temporal gyrus	46	−62	−12	6.38	8.7	52
L inferior occipital gyrus	−26	−92	−8	6.02	8.22	34
R middle occipital gyrus	32	42	34	6.07	8.14	145
R middle frontal gyrus	34	50	20	6.34	8.13	102
L precuneus	−20	56	34	6.17	7.99	24
L insula	−54	18	0	6	7.88	156
R postcentral gyrus	38	−36	66	6.34	7.82	30
R supramarginal gyrus	52	−30	52	5.92	7.71	41
R superior frontal gyrus	32	−2	60	5.81	7.46	43
L superior occipital gyrus	−26	−74	34	5.68	6.64	31
L putamen	−22	−2	8	5.60	6.44	50
R middle cingulate gyrus	4	16	38	5.61	6.25	14
L anterior cingulate gyrus	−8	32	20	5.52	6.08	15
L postcentral gyrus	−60	−12	20	5.60	6.06	30
**Main effect of group PIGU < HC (*k* > 20)**						
R calcarine	18	−80	14	6.77	12.59	247
L calcarine	−8	−92	−6	7.32	11.95	82
L cuneus	−4	−92	8	7.11	11.62	57
R middle occipital gyrus	38	−86	14	6.43	9.35	64
L fusiform gyrus	−36	−78	−18	6.72	9.20	23
R inferior occipital gyrus	26	−94	−6	6.29	8.41	18
R fusiform gyrus	36	−52	−24	6.11	7.56	4
R lingual gyrus	16	−68	−8	7.34	5.88	37
L lingual gyrus	−8	−60	−4	6.72	5.66	16
L superior occipital gyrus	−14	−86	20	5.59	6.14	17
**Main effect of condition**						
L precuneus	−2	−58	26	6.38	9.30	484
L anterior cingulate gyrus	−6	38	−12	5.93	8.05	307
L postcentral gyrus	−52	−12	52	5.31	5.46	9
L anterior cingulate gyrus	0	26	16	5.29	5.4	4
R inferior temporal gyrus	46	−58	−10	5.21	5.21	2
Group * condition interaction						
No significant activation						

**L, left; R, right*.*

**FIGURE 1:**
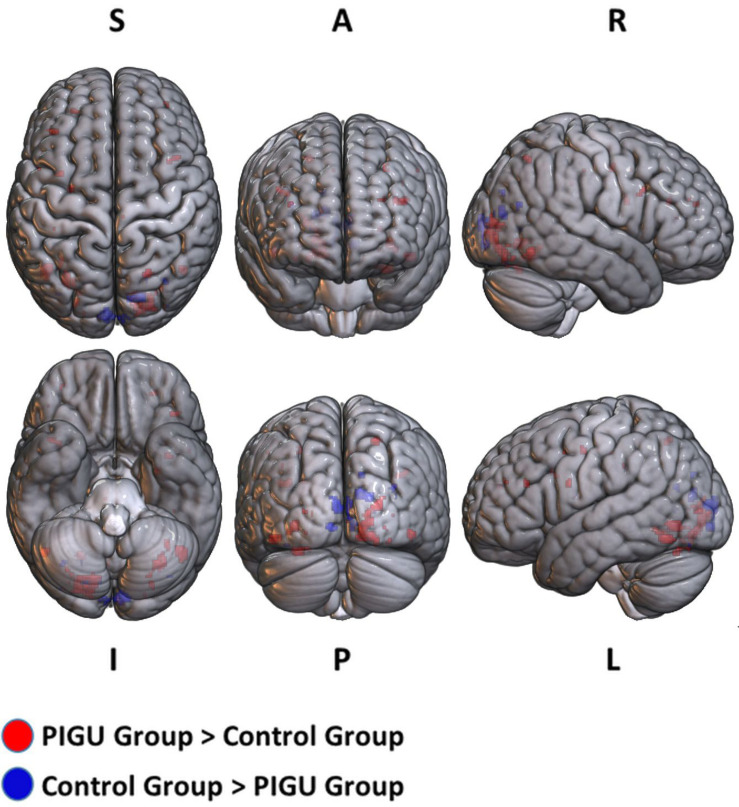
3D volume rendered images of whole brain activation during positive emotional valence conditions in the PIGU group (red regions) and control group (blue regions). Activation threshold is set at pFWE < 0.05.

The between-group whole-brain activation in the PIGU group compared to the control group condition (PIGU group > Control group) and the control group compared to the PIGU group condition (Control group > PIGU group) while viewing the positive emotional valence pictures are shown in Supplementary Tables 5, 6. All these regions showed significant activation at pFWE < 0.05 (see Figure 1). When testing for the positive emotional valence cues, the PIGU group (PIGU group > control group) exhibited increased activation in the superior occipital gyrus (SOG), left MOG, left inferior occipital gyrus (IOG), right fusiform gyrus, right lingual gyrus, left IFG, left MFG, left precentral gyrus, left inferior operculum frontal gyrus, parietal lobe, right superior and left inferior parietal lobule, postcentral gyrus, cingulate cortex, left precuneus, right IFG, right inferior temporal gyrus (ITG), left cuneus, and left precentral gyrus. Additionally, subjects in the PIGU group (Control group > PIGU group) exhibited lower activation in the calcarine cortex, right cuneus, left lingual, and right MOG in response to the positive emotional valence cues.

**FIGURE 2:**
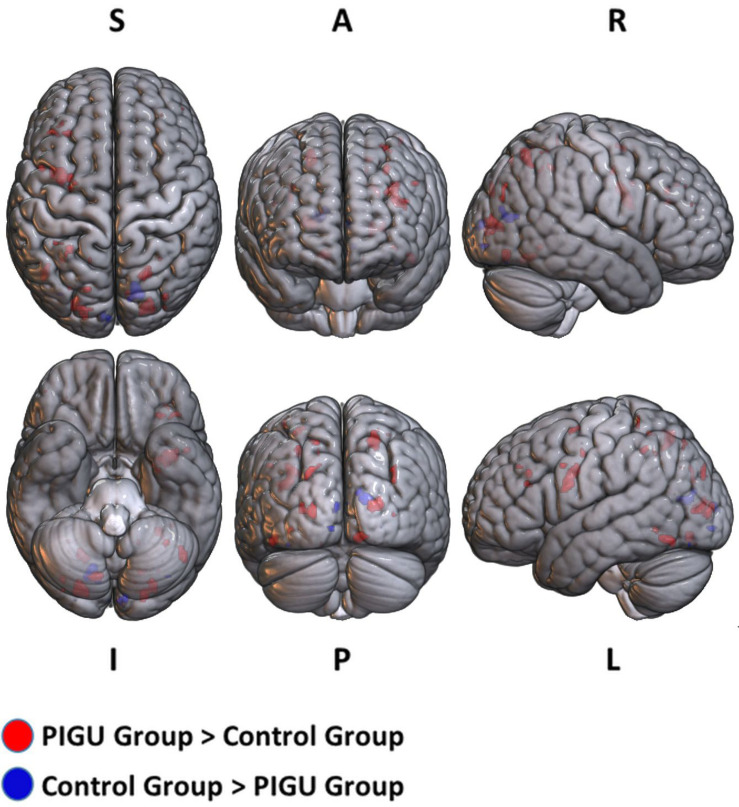
3D volume rendered images of whole brain activation during negative emotional valence conditions in the PIGU group (red regions) and control group (blue regions). Activation threshold is set at pFWE < 0.05.

The between-group whole-brain activation in the PIGU group compared to the control group (PIGU group > Control group) and control group compared to the PIGU group (Control group > PIGU group) when presented with negative emotional valence cues are shown in the Supplementary Tables 7, 8. All these regions showed significant activation at pFWE < 0.05 (see Figure 2). When processing the negative emotional valence cues, the PIGU group as compared to the control group (PIGU group > control group) exhibited increased activations predominantly in regions of the PFC. Specifically, activations were noted in the SOG, left MOG, left IOG, right fusiform gyrus, right lingual gyrus, left IFG, left MFG, left precentral gyrus, postcentral gyrus, left inferior operculum frontal gyrus, parietal lobe, right superior and left inferior parietal lobule, and the right MOG. When testing for the contrast Control group > PIGU group, the PIGUs exhibited lower activations in the calcarine cortex, bilateral cuneus, left lingual, left fusiform gyrus, right IOG, and right MOG in response to the negative emotional valence cues.

Within-group analysis among the PIGU group revealed that the contrast for negative emotional valence > positive emotional valence cues elicited significant activations in the left Prec node of the dopaminergic reward network. Conversely, there were no surviving nodes detected when testing for negative emotional valence > positive emotional valence images among the HC group.

**FIGURE 3:**
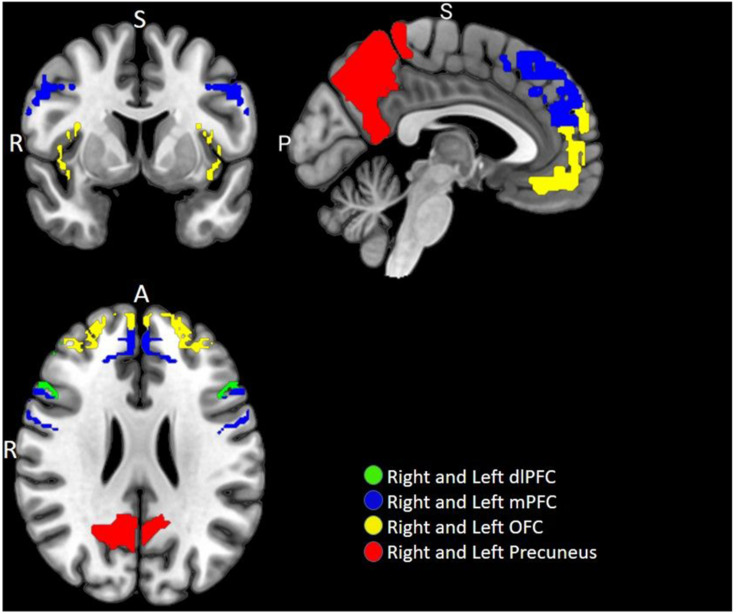
Multi-slice axial view of brain functional image subtraction in PIGU > Control group contrast, when presented with negative emotional valence cues.

*A priori* defined ROI analyses were performed to test the hypothesis pertaining to the neural responses toward negative emotional valence cues (see Table 4). Data shown are from the activation map for the PIGU group > Control group at *p* < 0.001 (FWE uncorrected). Left ACC and bilateral amygdala did not survive thresholds for multiple comparison in this corresponding analysis. Voxels that survived the final inclusion threshold of *p* < 0.001 are shown in Figure 3. Table 5 shows the average PSC for each *a priori* defined ROI. A Mann–Whitney *U* test was performed to test whether the mean PSC across subjects was significantly different (see Table 5). Between-group comparisons (PIGU group > HC group) showed no significant PSC in cue-induced brain activations at selected ROIs when presented with the negative emotional valence condition when corrected for multiple comparisons (Mann–Whitney *U* test; *p* < 0.004, Bonferroni correction made for multiple comparisons). Prior to correction for multiple comparisons, the left precuneus [peak = −12 –58 50; *p*(uncorrected) = 0.033] and the right precuneus [peak 16 −64 48; *p*(uncorrected) = 0.037] showed significant differences in the PSC between the two groups. Whereas ROI discriminating the ECN revealed a significantly larger PSC at the left dlPFC and left mPFC among the PIGU (Table 4) (see Figure 3).

**TABLE 4:** *A priori* defined ROI analysis in PIGU and HC Groups when presented with negative emotional valence cues.

			MNI coordinates			
Regions	Peak *T*	Mean T	*X*	*Y*	*Z*	*P*
L dlPFC	5.65	5.01	−36	4	28	<0.001
L mPFC	5.35	4.79	−50	6	36	<0.001
L OFC	4.51	4.14	−30	46	28	<0.001
L precuneus	5.14	4.63	−12	−58	50	<0.001
R dlPFC	5.30	4.75	32	42	34	<0.001
R mPFC	5.56	4.94	46	46	22	<0.001
R OFC	5.00	4.52	34	58	20	<0.001
R precuneus	4.68	4.28	16	−64	48	<0.001
R ACC	3.43	3.26	10	28	28	0.001
Amygdala (R/L)	No voxel survived					
L ACC	No voxel survived					

**R dlPFC/L dlPFC, right/left dorso-lateral prefrontal cortex; L mPFC/R mPFC, left/right medial prefrontal cortex; L OFC/R OFC, left/right orbito-frontal cortex; R ACC/L ACC, right/left anterior cingulate cortex*.*

**TABLE 5:** Average percent change of fMRI signal at selected ROIs in PIGU and HC groups when presented with negative emotional valence cues (Bonferroni correction for multiple comparisons).

	PSC (Mean ± *SD*)		
Regions	PIGU group	HC group	*p*-value
L dlPFC	0.499 ± 0.327	0.275 ± 0.195	0.067
L mPFC	0.643 ± 0.387	0.378 ± 0.261	0.116
L OFC	0.416 ± 0.473	0.216 ± 0.249	0.345
L precuneus	0.225 ± 0.233	0.035 ± 0.169	0.033
R dlPFC	0.372 ± 0.397	0.134 ± 0.273	0.161
R mPFC	0.698 ± 0.445	0.383 ± 0.396	0.074
R OFC	0.803 ± 0.661	0.452 ± 0.420	0.202
R precuneus	0.463 ± 0.364	0.162 ± 0.233	0.037
R ACC	0.224 ± 0.214	0.177 ± 0.188	0.744

**Mann–Whitney U test with Bonferroni correction for multiple comparisons at p < 0.004*.*

To assess the size and direction of the linear relationship between PSC during negative emotional valence Instagram cues condition and the severity of PIGU based on the IGAT scores, a Spearman’s Rho (ρ) was computed (see Supplementary Tables 9, 10). A negative and moderate correlation was observed between the PSC at the right mPFC with the IGAT scores of the PIGU subjects when corrected for multiple comparisons [*r* = −0.777, (*p* < 0.004, two-tailed)]. There were, however, no significant correlations between IGAT scores and the PSC at the selected ROIs in the HC group.

## Discussion

The advantage of the present study is that it gives a distinct dimension in understanding the neurobiology of social media addiction, particularly the consequences of Instagram use among young adults. This study is also pertinent in giving evidence-based medicine considering that prior experiments using questionnaire-based responses only studied information that assessed the reflective cognitive judgment, whereas fMRI-task behavior can clearly illustrate automatic emotional judgments (Takami and Haruno, 2018).

Although most previous studies were able to demonstrate the activation of the dopaminergic reward network (Sherman et al., 2016; Wegmann et al., 2018); however, there was a previous fMRI study which was unable to prove a lack of inhibitory control was exerted by the ECN among SNA users potentially at risk of having social media addiction. The latter study postulated this to arise from the small sample size, moderate symptoms of addiction among the research participants, and potential differences between Facebook addiction and other types of addictions (Turel et al., 2014).

In this study, negative emotional valence Instagram cues were used as rewarding stimuli to induce a feeling of ‘craving’ among the PIGU group. As hypothesized, the PIGU group responded favorably to the perceived rewarding stimuli during the task-based fMRI cue-reactivity study. The PIGU subjects demonstrated increased activation in the Prec compared with the healthy controls when presented with addiction-specific cues. Precisely, activation was observed in the Prec for negative emotional valence > positive emotional valence Instagram-themed cues among the PIGU group, but not in the control group, before correction for multiple comparisons was made. The activation of the Prec, within the context of the more extensive cortico-striatal reward network, is postulated to reflect its integration and relay of its non-drug-specific role in cue-reactivity that extends from the visual system to the systems involved in motivated behavior (Courtney et al., 2014). Additionally, the activation of the Prec in response to the cue-reactivity paradigm has been reported as a response toward creating a flow of reward-related information from the visual cortex to the limbic system (Engelmann et al., 2012). This observation is similar to the regional activation elicited by rewarding stimuli among subjects with online PG and internet gaming disorders (Dong and Potenza, 2016; Starcke et al., 2018).

In response to positive emotional valence Instagram cues, subjects in the PIGU group compared to the control group also exhibited activation of the ventral ACC and the left Prec. We postulate that this is likely due to the increased focus toward the cues being a ‘positive reward’ for the PIGU, i.e., depicting that the users were paying attention because they could relate past experiences with the cues. Hence, the positive emotional valence Instagram cues, being a type of mildly stimulating reward, could induce activations in regions of the cingulate cortex. Furthermore, the ventral ACC is known to be involved in the processing of emotional responses toward a stimulus (Vogt and Nicola, 2012; Chun et al., 2018).

As hypothesized, the negative emotional valence cues caused intense activations of the dopaminergic reward system among the PIGU compared to control subjects. This is indicative of the inherent nature of social media addicts who tend to have cravings toward ‘risky’ cues. Similar results have been observed in studies pertaining to drug-related cues, and pathological risk-taking cues, which can also ‘hijack’ the brain’s reward system (Koob and Volkow, 2009). Additionally, the PIGU subjects were hypersensitive to risk-taking behavior, which is evident by the increased activations of the Prec. Regional activations have occurred in the left Prec among the PIGU group when presented with negative emotional valence > positive emotional valence conditions, which can be considered as a biomarker for detecting Instagram addiction. The preferential Prec activation among social media addicts in response to cue-reactivity paradigms is in line with previous literature related to internet gaming addiction and nicotine addiction (Courtney et al., 2014; Ding et al., 2014). Hence, the activation of the Prec among the PIGU subjects is explained by the fact that it is involved in the modulation of exteroception, i.e., the processing of external stimuli, assigning salience to the stimuli, as well as adapting and responding to the habit-forming stimuli (DeWitt et al., 2015).

Conversely, we observed a relative activation of the executive control network among the PIGU compared to the control group when presented with addiction-specific stimuli, namely in the dlPFC, mPFC, and OFC. The mPFC is involved in memory, reward-guided learning, and in decision-making on risks and rewards (Rushworth et al., 2011). A non-significant activation of the ECN was noted in the PIGU when presented with the negative emotional valence cues are indicative of dysfunctional control in the PFC of the PIGU subjects. Controversies surround the PFC even though its function is known to be highly integrated and flexible, i.e., multiple regions may be involved in performing a task. Nevertheless, specific regions have been implicated in responding to external stimuli (Goldstein and Volkow, 2011). In particular, the dlPFC has been implicated in top-down control and meta-cognitive functions. In contrast, the ventral mPFC, which includes the subgenual ACC and the medial OFC is involved in emotion regulation and assigning incentive salience to drugs and drug-related cues (Goldstein and Volkow, 2011).

Nevertheless, there was a significant negative correlation between the right mPFC activation with the IGAT score, which indicated that some areas of the ECN became deactivated with the increasing severity of Instagram addiction. It is known that the frontal lobe is involved in decision-making and cognitive functioning, and specifically, the mPFC is a critical brain region involved in decision-making and decides whether to ‘approach’ or to ‘avoid’ a stimulus (Euston et al., 2012). This implies that the PIGU group had reduced mPFC activity due to their impaired decision-making ability. Despite these preliminary findings, caution needs to be exercised in interpreting the data due to the small sample size and correlative nature of this study.

The control group, however, exhibited a normal inhibitory response to negative emotional valence Instagram cues, as observed by the increased activation of the mPFC in this group. In summary, the inability of the PIGU group to regulate their executive control toward ‘risky’ Instagram posts with a high number of likes may lead them to seek the rewarding stimuli to satisfy their cravings continuously.

A clear picture emerges when comparing the cues selected in this fMRI paradigm as compared to prior studies that have incorporated various types of risky behavior to evaluate the ECN among Instagram addicts. Particularly in Asian cultures, binge drinking alcohol, publicly smoking cigarettes, online sexual content, and pornography are considered taboo. Thus, the inherent conservativeness of the Asian culture may explain why certain types of risky behaviors such as taking selfies from heights and dangerous locations, texting while driving and risqué interpersonal situations can be prevalently seen in their Instagram posts (Zhou et al., 2017). Furthermore, it is a known fact that impulsive people use smartphones as a gratifying means to engage in activities with no forethought about the consequences of their actions (Mitchell and Potenza, 2014). Thus, it can be postulated that impulsivity triggers the development of behavioral addictions (Cuzen and Stein, 2014).

Furthermore, a study among a group of university students in China found that mobile phone dependency was associated with impulsive behavior (Mei et al., 2018). The attractiveness of the photo-sharing features of Instagram, together with its easy availability and instant access, increases the addictiveness among young adults by providing them with a pleasurable distraction. Thus, problematic users fail to think about the negative consequences of their actions, further enforcing the failure of the ECN to inhibit addictive behavior.

Furthermore, a recent study by Jacobs-Brichford et al. (2019) postulated that the exposure to an addictive substance, in their case exposure to cannabinoids, during the adolescent period leads to impairment of the excitatory-inhibitory signal balance in the mPFC that can persist into adulthood (Jacobs-Brichford et al., 2019). Similarly, we postulate that although our subjects are young adults who reveal abnormal dlPFC and mPFC activity in response to risky cues, they had been using Instagram since their adolescent age, which may have caused impairment in the maturation process of their executive control network. We recommend future longitudinal studies to follow-up on addicted individuals from adolescence to adulthood to better understand the long-term effects of social media addiction.

The findings in this study provide an objective assessment of the behaviors that occur in the online and offline environments. We can gain novel insights regarding the neural systems that support PIGU behavior.

Lastly, it is essential to note that the increased number of times that the PIGU group selected the Instagram-Go cues and the longer duration of response time to Instagram-Go cues compared to No-go cues may manifest from the habitually retrievable implicit associations caused by a hyperactive dopaminergic reward system. Nevertheless, the appropriateness of the use of the addiction vs. problematic use is also debatable due to the lack of consensus among various diagnostic criteria and behavioral tests. The presence of a negative correlation between the activations in the mPFC and the severity of PIGU, albeit non-significant, warrants further research with larger sample sizes as well as improvements in the design of the behavioral tests and addiction scales. There is also a need to explore the effects of age, gender, socio-economic, and cultural differences that may affect the nuances of social media addiction.

### Limitations and Future Recommendations

This study had some limitations in terms of small sample size. Given that this is a pilot, in which the main aim was to provide a proof of concept for quantifiable parameters to assess social networking addiction, we propose that future studies perform a power of study assessment and recruit larger sample sizes. Additionally, in view of similar motivations pertaining to seeking likes, which is a common feature in many social networking sites, thus, our findings may have similarities that could be generalized with the neurobiology of other SNA addictions. Another limitation of this study is that there are times that fMRI acquisitions may experience motion artifacts, as we too have experienced in 6 out of our initial 36 fMRI study participants. We had to discard those scans as they would have negatively affected the overall results of finding significant PSC at the selected ROIs. Therefore, the impact of this can be minimized in future studies by increasing the sample size. Furthermore, the image valence and the number of likes associated with the pictures are not independently manipulated, making it difficult to distinguish the effects of these two factors. These considerations need to be made by future researchers seeking to further elucidate the neurobiological underpinnings of social media addictions.

Nevertheless, considering that a significant number of previous publications have resorted to monetary gain paradigms to evaluate their cue-induced reactivity experiments, this type of fMRI study is a novel addition to addiction-specific cues. Hence, we propose that future studies design improved paradigms customized to different SNA platforms, which can enable more robust activations of the dopaminergic reward system together with more substantial percentage signal changes in the executive control network. Longitudinal studies are also proposed to elucidate the neural mechanisms of addiction that evolve with age.

We also recommend an event-based fMRI paradigm, considering the tasks can be displayed for shorter durations, which can potentially elicit more significant regional neuronal activations. This study did not evaluate the subjects for possible attention deficit hyperactivity disorder (ADHD), which has been implicated to be exacerbated in subjects who spend prolonged time on their smartphones. Future researchers may also consider a more thorough neuropsychiatric assessment prior to recruiting subjects as this can better define the criteria for SNA addiction.

In summary, it is crucial to design an interface-specific cue-reactivity paradigm because it can be more accurate in eliciting ‘cravings’ in the addicts, thus more robust activations can be detected on fMRI. Consequently, fMRI studies can objectively assess the neural pathways of social media addiction and elicit an objective response, which can guide personalized treatment plans and modify follow-up protocols.

## Conclusion

Addiction-specific Instagram-themed cue-induced reactivity tasks can illustrate the neurobiology of addiction among problematic Instagram users. Activations of the dopaminergic reward system and deactivation of the executive control network indicate converging neuropathological pathways between Instagram addiction and other types of addictions.

## Data Availability Statement

The raw data supporting the conclusions of this article will be made available by the authors, without undue reservation, to any qualified researcher.

## Ethics Statement

The studies involving human participants were reviewed and approved by Universiti Putra Malaysia Ethics Committee (UPM/TNCPI/RMC/1.4.18.2/JKEUPM). The patients/participants provided their written informed consent to participate in this study.

## Author Contributions

SS and LL helped in securing financial support for this project. SS, SF, and CM designed and conceptualized the study. NN, HS, AR, MM, RT, and HK made substantial contributions to methods, data collection, data analysis, and data interpretation. SS and NN wrote the manuscript. SS, ER, SH, and MM read and approved the final manuscript. All authors contributed to the article and approved the submitted version.

## Conflict of Interest

RT was employed by Aerobe Inc. Singapore. The remaining authors declare that the research was conducted in the absence of any commercial or financial relationships that could be construed as a potential conflict of interest.

## Footnotes

1 http://www.fil.ion.ucl.ac.uk/spm/software/spm12/

2 https://www.nitrc.org/projects/wfu_pickatlas/

3 https://sourceforge.net/projects/marsbar/
