# Supplementary material for: Cue-Reactivity Among Young Adults With Problematic Instagram Use in Response to Instagram-Themed Risky Behavior Cues: A Pilot fMRI Study
Source: Front Psychol. 2020 Nov 2;11:556060. doi: 10.3389/fpsyg.2020.556060 (PMC7667047; doi:10.3389/fpsyg.2020.556060)
Supplement: Supplementary file 1 [file Data_Sheet_1.docx]

Supplementary Material

# SUPPLEMENTARY DATA

**Supplementary file 1**

**Diagnostic criteria by Lin et al. (2017)**


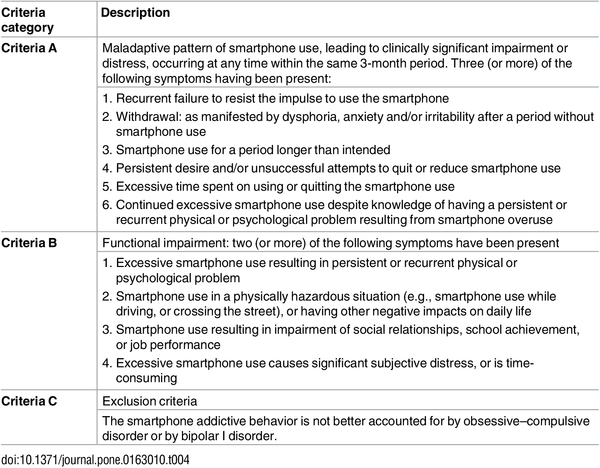


**Supplementary file 2**

**SAS-M questionnaire – adopted from Ching et al. (2015)**


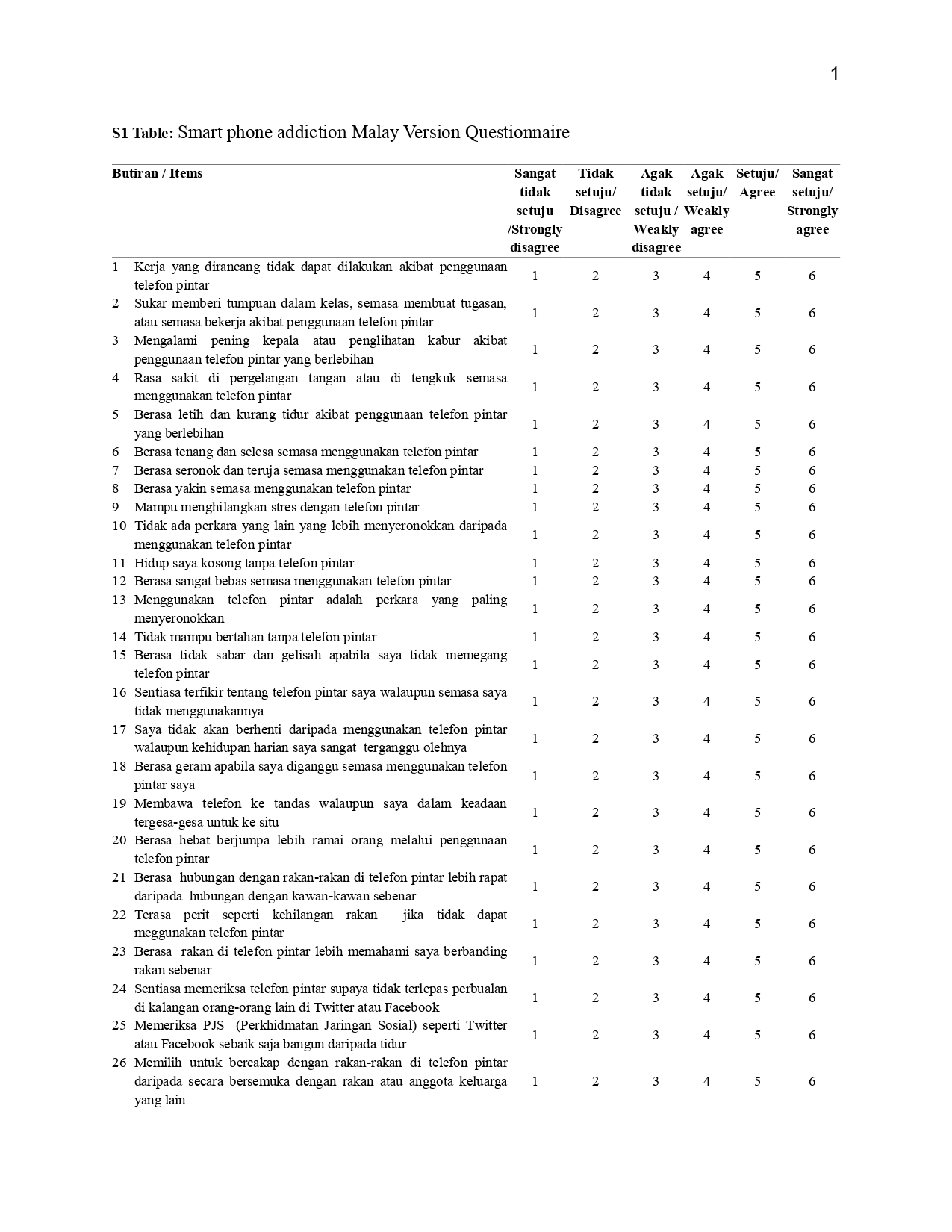


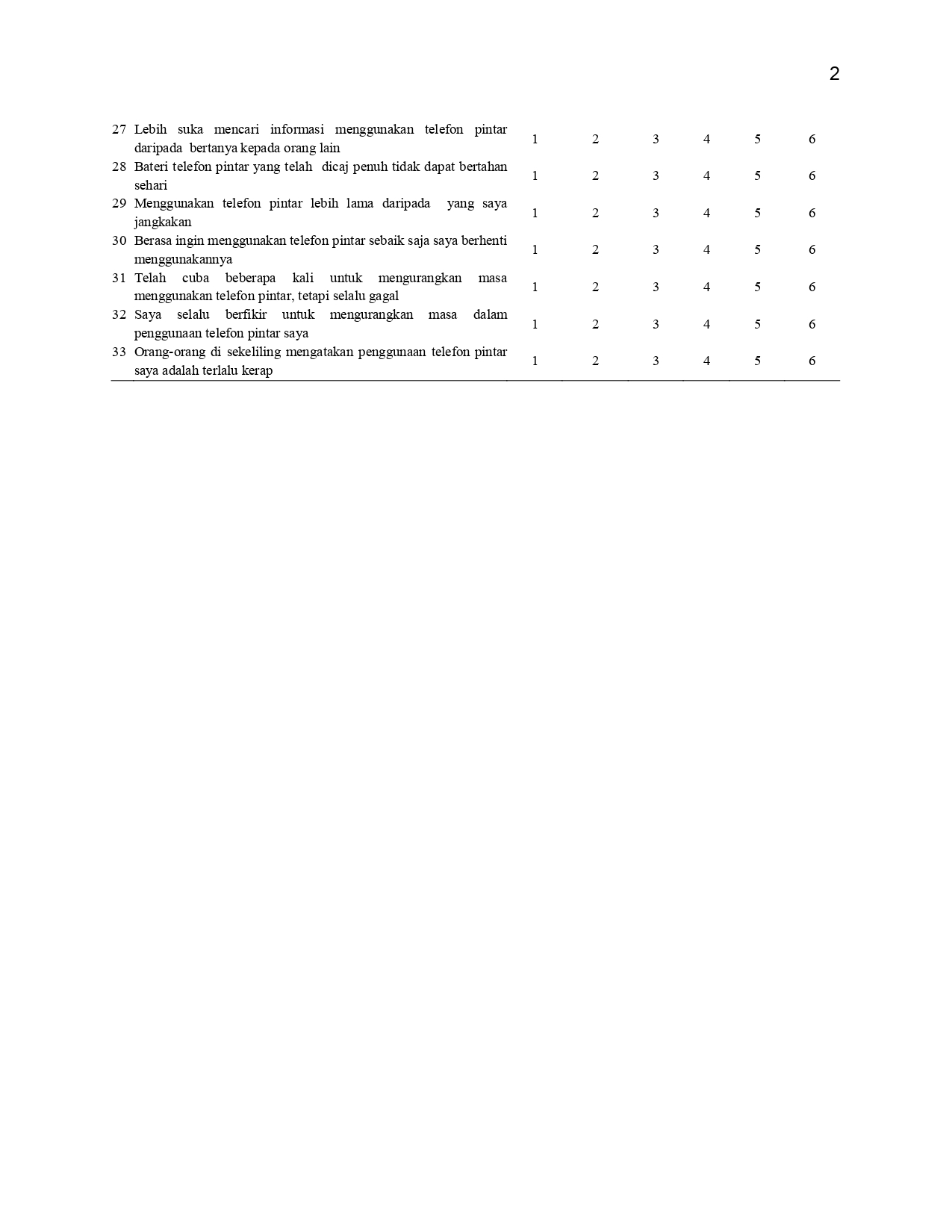


**Supplementary file 3**

**Modified IGAT questionnaire – modified from Pawlikowski et al. (2013)**


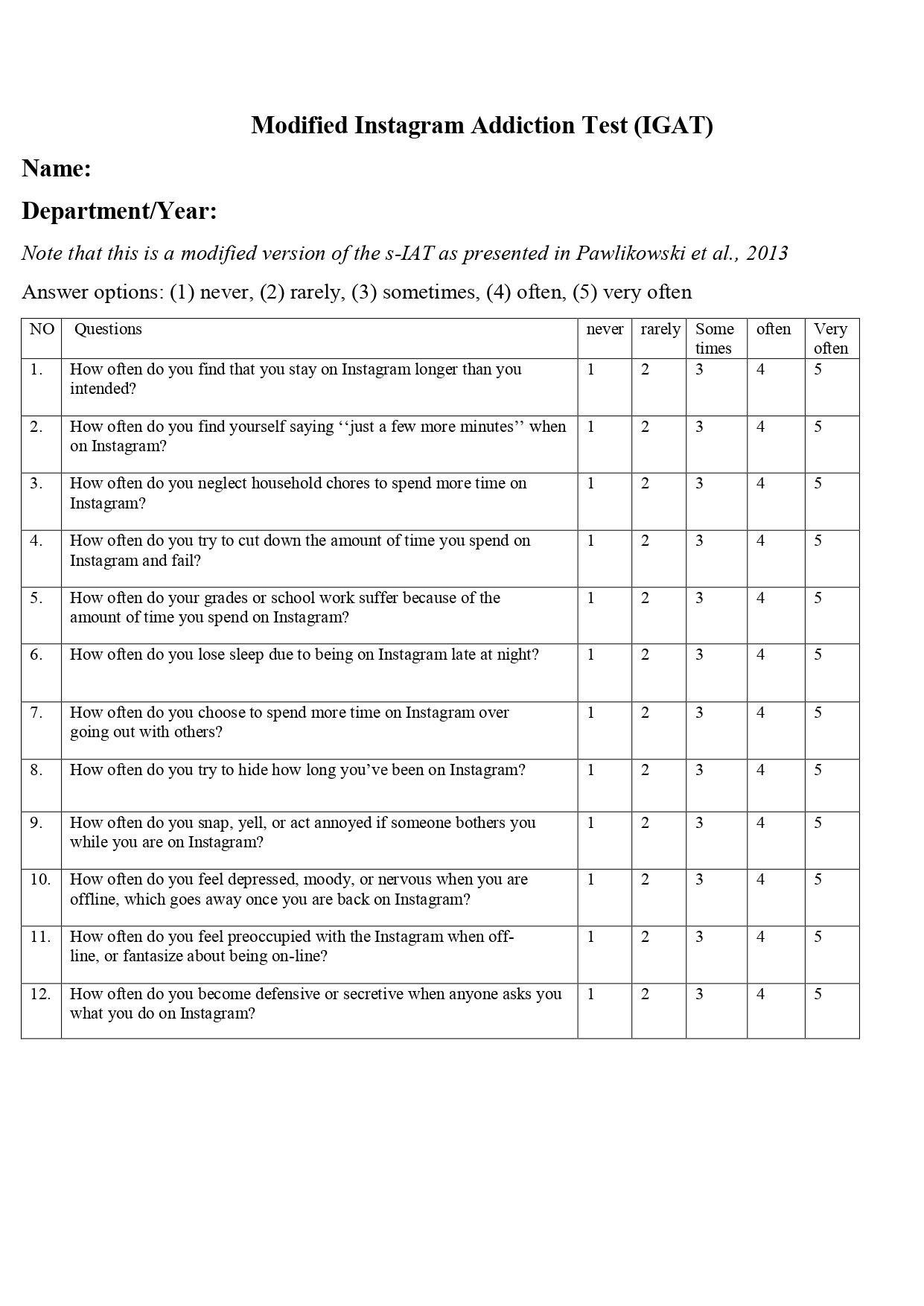

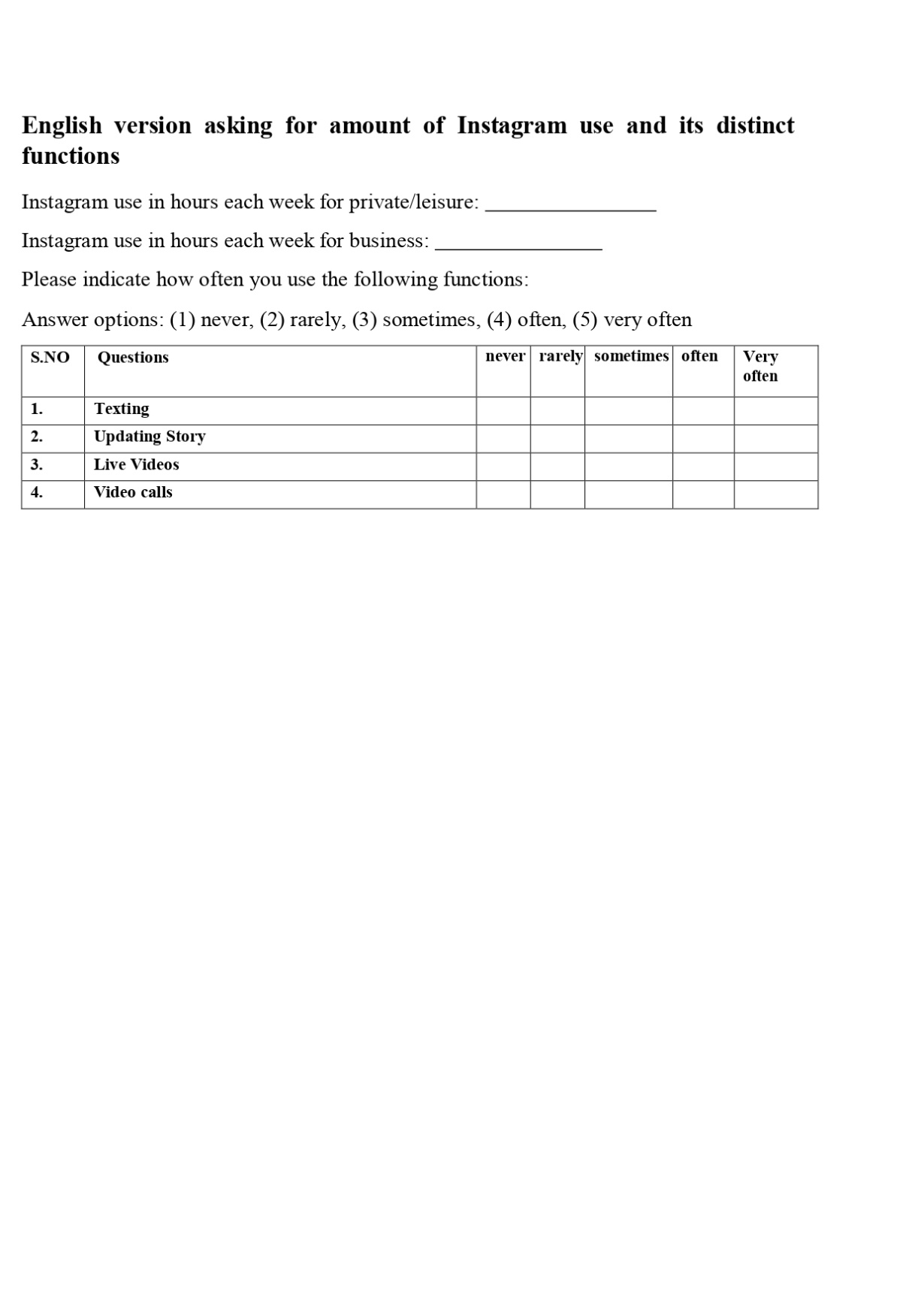


# SUPPLEMENTARY FIGURES


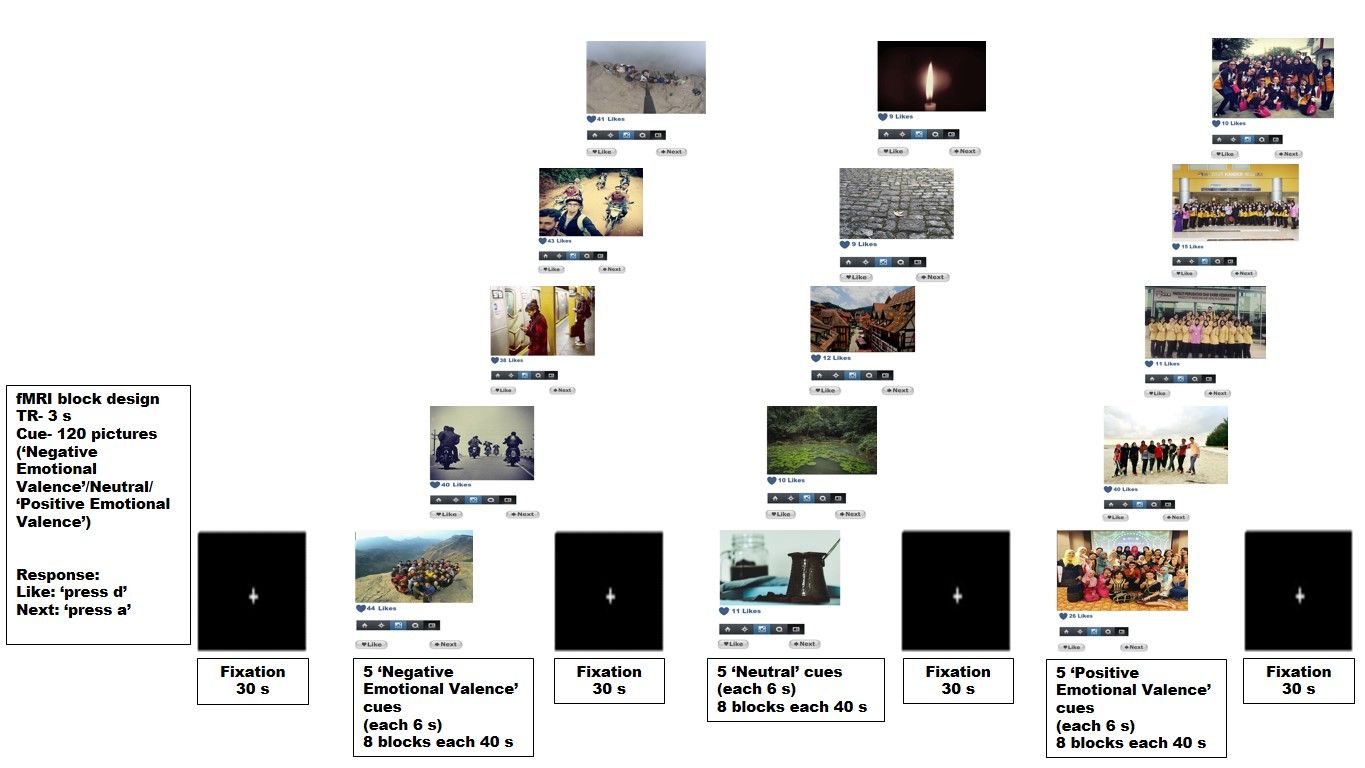


**Supplementary Figure S1. fMRI cue-reactivity paradigm to assess neural response of PIGU towards Instagram-based rewards**

# SUPPLEMENTARY TABLES

**Supplementary Table S1. Scores attributed by the participants for various dimensions of the SAS-M questionnaires**

| **Components** | **Mean ± SD** | |
| --- | --- | --- |
|  | **PIGU group** | **Control group** |
| **Cyberspace-oriented relationship**  **(Questions 19,20,21,22,23,24 and 26)** | 22.667 ± 6.98 | 9.73 ± 2.66 |
| **Daily life disturbance**  **(Questions 1,2,3,4,5 and 33)** | 26.8 ± 5.92 | 12.4 ± 4.17 |
| **Primacy**  **(Questions 10,11,12,13 and14)** | 17.867 ± 4.52 | 8.8 ± 3.75 |
| **Overuse**  **(Questions 25,27,28,29,30,31 and 32)** | 31.4 ± 5.12 | 15.667 ± 4.24 |
| **Positive anticipation**  **(Questions 6,7,8 and 9)** | 17 ± 2.33 | 11.6 ± 5.78 |
| **Withdrawal**  **(Questions 15,16,17 and 18)** | 14.067 ± 5.09 | 6.2 ± 2.62 |

**Supplementary Table S2. Response time and response type based on cue-reactivity towards different conditions among the PIGU and healthy control groups**

|  | **Response Time (ms)** | | | **Number of Response Type (choose ‘Like’ or choose ‘Pass’)** | | | | |
| --- | --- | --- | --- | --- | --- | --- | --- | --- |
|  | **‘Positive Emotional Valence’ Images** | **‘Negative Emotional Valence’ Images** | **Neutral Images** | **‘Negative Emotional Valence’ Press Like** | **‘Negative Emotional Valence’ Press Pass** | **Neutral Press Like** | **Neutral Press Pass** | |
| **PIGU** |  |  |  |  |  |  |  | |
| 1 | 46.363 | 54.853 | 51.72 | 4 | 36 | 26 | | 14 |
| 2 | 66.23 | 63.73 | 58.24 | 2 | 38 | 19 | | 21 |
| 3 | 102.121 | 99.57 | 85.72 | 21 | 19 | 25 | | 15 |
| 4 | 53.55 | 57.332 | 71.567 | 6 | 34 | 34 | | 6 |
| 5 | 117.129 | 148.883 | 133.038 | 26 | 14 | 34 | | 6 |
| 6 | 98.354 | 127.302 | 98.73 | 15 | 25 | 18 | | 22 |
| 7 | 78.917 | 105.354 | 97.98 | 12 | 28 | 14 | | 26 |
| 8 | 37.593 | 55.332 | 60.903 | 26 | 14 | 28 | | 12 |
| 9 | 38.925 | 63.831 | 44.843 | 22 | 18 | 26 | | 14 |
| 10 | 90.983 | 76.741 | 61.919 | 23 | 17 | 10 | | 30 |
| 11 | 60.836 | 77.646 | 80.699 | 13 | 27 | 16 | | 24 |
| 12 | 69.728 | 86.983 | 82.784 | 20 | 20 | 5 | | 35 |
| 13 | 98.673 | 106.47 | 94.62 | 6 | 34 | 19 | | 21 |
| 14 | 20.280 | 30.630 | 30.290 | 25 | 15 | 28 | | 12 |
| 15 | 69.977 | 82.48 | 75.218 | 16 | 24 | 22 | | 18 |
| **Mean** | 69.977 | 82.475 | 75.218 | 17.307 | 24.2 | 21.6 | | 20.307 |
| **SD** | 27.792 | 30.944 | 25.585 | 7.909 | 8.334 | 8.304 | | 7.134 |
| **HC** |  |  |  |  |  |  | |  |
| 1 | 53.433 | 61.549 | 64.682 | 30 | 10 | 9 | | 31 |
| 2 | 46.712 | 77.462 | 52.634 | 0 | 40 | 20 | | 20 |
| 3 | 64.742 | 77.2 | 68.706 | 7 | 33 | 33 | | 7 |
| 4 | 59.027 | 43.718 | 46.726 | 0 | 40 | 6 | | 34 |
| 5 | 68.504 | 94.574 | 82.541 | 21 | 19 | 18 | | 22 |
| 6 | 51.37 | 79.156 | 58.48 | 13 | 27 | 10 | | 30 |
| 7 | 52.052 | 56.464 | 70.102 | 0 | 40 | 5 | | 35 |
| 8 | 56.439 | 62.422 | 72.258 | 5 | 35 | 30 | | 10 |
| 9 | 71.129 | 83.433 | 92.568 | 24 | 16 | 27 | | 13 |
| 10 | 49.15 | 59.639 | 48.337 | 0 | 40 | 13 | | 27 |
| 11 | 52.606 | 69.587 | 80.215 | 11 | 29 | 24 | | 16 |
| 12 | 42.825 | 49.098 | 51.245 | 22 | 18 | 12 | | 28 |
| 13 | 60.271 | 87.444 | 73.325 | 1 | 39 | 8 | | 32 |
| 14 | 52.62 | 65.65 | 57.94 | 5 | 35 | 26 | | 14 |
| 15 | 63.122 | 76.018 | 70.621 | 13 | 27 | 19 | | 21 |
| **Mean** | 56.266 | 69.560 | 66.025 | 10.133 | 29.866 | 18.142 | | 22.666 |
| **SD** | 8.072 | 14.341 | 13.424 | 10.07 | 10.07 | 8.899 | | 9.131 |
| **pvalue** | 0.085 | 0.158 | 0.228 | **0.048*** | 0.104 | 0.289 | | 0.458 |

*Significant at p < 0.05

**Supplementary Table S3. Brain Activation in Response to ‘Neutral’ Pictures in the PIGU Group as Compared to Control group (PIGU Group > Control Group)**

| **PIGU Group > Control Group**  **(‘Neutral’) ; at pFWE<0.05** | | | | | | |
| --- | --- | --- | --- | --- | --- | --- |
|  |  |  |  | **Talairach coordinates** | | |
| **Regions** | **Voxel** | **Peak t** | **Mean t** | **X** | **Y** | **Z** |
| L IFG | 38 | 7.27 | 5.76 | -40 | 32 | 22 |
| LSOG | 13 | 7.02 | 6.00 | -20 | -88 | 32 |
| L Inferior Operculum Frontal | 37 | 6.93 | 5.84 | -38 | 2 | 24 |
| L IOG | 20 | 6.92 | 5.83 | -46 | -64 | -14 |
| L MOG | 19 | 6.46 | 5.73 | -20 | -92 | 6 |
| L MFG | 46 | 6.45 | 5.66 | -26 | 44 | 24 |
| L Precentral Gyrus | 41 | 6.10 | 5.53 | -48 | 2 | 36 |
| R SFG | 14 | 6.09 | 5.43 | 34 | -4 | 62 |
| R SOG | 7 | 6.08 | 5.62 | 26 | -88 | 10 |
| R Lingual Gyrus | 7 | 5.89 | 5.45 | 10 | -78 | -12 |
| R Rolandic Operculum | 5 | 5.74 | 5.58 | 64 | 8 | 10 |
| R Postcentral Gyrus | 7 | 5.70 | 5.38 | 38 | -36 | 66 |
| R Fusiform Fyrus | 7 | 5.56 | 5.40 | 30 | -76 | -16 |
| R MOG | 2 | 5.52 | 5.39 | 32 | -80 | 22 |
| R MFG | 1 | 5.45 | 5.33 | 26 | 48 | 32 |
| R MCC | 1 | 5.37 | 5.29 | 40 | 12 | 30 |
| L Superior Parietal Lobe | 2 | 5.33 | 5.28 | -32 | -52 | 58 |
| L Postcentral Gyrus | 2 | 5.21 | 5.21 | -40 | -42 | 62 |
| R Superior Parietal Lobe | 2 | 5.18 | 5.17 | 32 | -54 | 56 |

L IOG: Left Inferior Occipital Gyrus; L SOG/R SOG: Left/Right Superior Occipital Gyrus; L IFG: Left Inferior Frontal Gyrus; L MOG/R MOG: Left/Right Middle Occipital Gyrus; R MCC: Right Middle Cingulate Gyrus; R MFG/L MFG: Right/Left Middle Frontal Gyrus; R SFG: Right Superior Frontal Gyrus.

**Supplementary Table S4. Brain Activation in Response to ‘Neutral’ Pictures in the Control Group as Compared to the PIGU Group (Control Group > PIGU Group)**

| **Control Group > Test Group**  **(‘Neutral’) ; at pFWE<0.05** | | | | | | |
| --- | --- | --- | --- | --- | --- | --- |
|  |  |  |  | **Talairach coordinates** | | |
| **Regions** | **Voxel** | **Peak t** | **Mean t** | **X** | **Y** | **Z** |
| L Calcarine | 70 | 9.14 | 6.7 | -4 | -94 | 8 |
| R Calcarine | 40 | 7.68 | 5.96 | 18 | -80 | 14 |
| R Middle Occipital Gyrus | 21 | 7.10 | 5.64 | 38 | -86 | 14 |

L: Left; R: Right

**Supplementary Table S5. Brain Activation in Response to ‘Positive Emotional Valence’ Pictures in the Test group as Compared to the Control group (PIGU Group > Control Group)**

| **PIGU Group > Control Group**  **(‘Positive Emotional Valence’) ; at pFWE<0.05** | | | | | | |
| --- | --- | --- | --- | --- | --- | --- |
|  |  |  |  | **Talairach coordinates** | | |
| **Regions** | **Voxel** | **Peak t** | **Mean t** | **X** | **Y** | **Z** |
| LIOG | 25 | 7.50 | 6.02 | -46 | -62 | -14 |
| L Fusiform Gyrus | 9 | 6.91 | 5.77 | -32 | -68 | -4 |
| R Orbito Inferior Frontal Gyrus | 28 | 6.77 | 5.70 | 40 | 12 | 30 |
| R SOG | 11 | 6.75 | 5.88 | 24 | -86 | 10 |
| R Lingual | 66 | 6.74 | 5.65 | 14 | -84 | -6 |
| R Superior Parietal Lobe | 19 | 6.57 | 5.69 | 22 | -64 | 52 |
| R ITG | 12 | 6.38 | 5.75 | 46 | -62 | -12 |
| Cerebellum Posterior Lobe | 12 | 6.26 | 5.57 | -28 | -70 | -22 |
| L IFG | 16 | 6.24 | 5.78 | -42 | 32 | 24 |
| L MOG | 6 | 6.14 | 5.50 | -26 | -82 | 4 |
| R IFG | 13 | 6.12 | 5.53 | 44 | 36 | 12 |
| L Inferior Operculum Frontal | 7 | 5.96 | 5.53 | -34 | 4 | 24 |
| R MCC | 11 | 5.96 | 5.52 | 4 | -26 | 44 |
| RMFG | 6 | 5.83 | 5.39 | 48 | 12 | 50 |
| L MFG | 13 | 5.71 | 5.42 | -26 | 44 | 24 |
| L SOG | 4 | 5.64 | 5.43 | -20 | -88 | 32 |
| L PCC | 1 | 5.52 | 5.35 | -6 | -74 | -16 |
| L Precentral Gyrus | 2 | 5.51 | 5.33 | -50 | 6 | 44 |
| R Orbito Medial Frontal Gyrus | 4 | 5.49 | 5.29 | 12 | 42 | -6 |
| L Cuneus | 1 | 5.48 | 5.48 | -14 | -88 | 36 |
| R Precentral Gyrus | 1 | 5.46 | 5.36 | 32 | 28 | 30 |
| L Precuneus | 1 | 5.43 | 5.32 | -32 | -2 | 8 |
| R Calcarine | 1 | 5.42 | 5.42 | 16 | -92 | 2 |
| R MOG | 1 | 5.36 | 5.36 | 34 | -86 | 2 |
| L Inferior Parietal Lobe | 1 | 5.34 | 5.26 | -40 | -34 | 36 |
| R Fusiform Gyrus | 1 | 5.32 | 5.31 | 28 | -76 | -6 |
| R Supra Marginal Gyrus | 2 | 5.30 | 5.25 | 64 | -28 | 40 |
| L Lingual Gyrus | 1 | 5.30 | 5.24 | -24 | -76 | -20 |

L IOG: Left Inferior Occipital Gyrus; L SOG/R SOG: Left/Right Superior Occipital Gyrus; R ITG: Right Inferior Temporal Gyrus; L IFG/R IFG: Left/Right Inferior Frontal Gyrus; L MOG/R MOG: Left/Right Middle Occipital Gyrus; R MCC: Right Middle Cingulate Gyrus; R MFG/L MFG: Right/Left Middle Frontal Gyrus; L PCC: Left Posterior Cingulate Cortex.

**Supplementary Table S6. Brain Activation in Response to ‘Positive-Emotional Valence’ Pictures in the Control Group as Compared to the PIGU Group (Control Group > PIGU Group)**

| **Control Group > PIGU Group**  **(‘Positive Emotional Valence’) ; at pFWE<0.05** | | | | | | |
| --- | --- | --- | --- | --- | --- | --- |
|  |  |  |  | **Talairach coordinates** | | |
| **Regions** | **Voxel** | **Peak t** | **Mean t** | **X** | **Y** | **Z** |
| L Calcarine | 144 | 10.87 | 6.87 | -4 | -92 | 8 |
| R Calcarine | 86 | 9.33 | 6.19 | 18 | -80 | 14 |
| R Lingual | 5 | 6.42 | 5.62 | 18 | -68 | 4 |
| R Middle Occipital Gyrus | 6 | 6.36 | 5.94 | 36 | -88 | 12 |
| R Cuneus | 10 | 6.02 | 5.45 | 12 | -80 | 28 |
| L Fusiform | 2 | 5.70 | 5.69 | -36 | -78 | -18 |
| L Lingual | 1 | 5.66 | 5.35 | 26 | -94 | -6 |
| R Superior Occipital Gyrus | 1 | 5.23 | 5.23 | 20 | -88 | 32 |

L: Left; R: Right

**Supplementary Table S7. Brain Activation in Response to ‘Negative Emotional Valence’ Pictures in the Test Group as Compared to Control Group (PIGU Group > Control Group)**

| **PIGU Group > Control Group**  **(‘Negative Emotional Valence’) ; at pFWE<0.05** | | | | | | |
| --- | --- | --- | --- | --- | --- | --- |
|  |  |  |  | **Talairach coordinates** | | |
| **Regions** | **Voxel** | **Peak t** | **Mean t** | **X** | **Y** | **Z** |
| L SOG | 42 | 8.25 | 6.05 | -22 | -88 | 32 |
| R SOG | 19 | 7.29 | 5.95 | 24 | -86 | 10 |
| L Inferior Operculum Frontal Gyrus | 34 | 7.15 | 5.84 | -38 | 2 | 22 |
| R Lingual Gyrus | 24 | 6.78 | 5.78 | 10 | -78 | -14 |
| L MOG | 16 | 6.61 | 5.78 | -20 | -90 | 4 |
| R MOG | 43 | 6.60 | 5.76 | 34 | -80 | 32 |
| R Superior Parietal Lobe | 52 | 6.52 | 5.67 | 22 | -64 | 52 |
| L IOG | 22 | 6.48 | 5.74 | -46 | -64 | -14 |
| L Superior Parietal Lobe | 11 | 6.35 | 5.62 | -18 | -76 | 50 |
| L Inferior Parietal Lobe | 16 | 6.30 | 5.59 | -30 | -48 | 46 |
| L Postcentral Gyrus | 13 | 5.97 | 5.52 | -40 | -42 | 64 |
| Parietal Lobe | 5 | 5.81 | 5.55 | 32 | -54 | 56 |
| L MFG | 8 | 5.76 | 5.43 | -30 | 30 | 30 |
| Cerebellum Posterior Lobe | 2 | 5.73 | 5.51 | -12 | -82 | -16 |
| R Inferior Operculum Frontal Gyrus | 4 | 5.73 | 5.46 | 46 | 16 | 36 |
| L Precentral Gyrus | 9 | 5.72 | 5.36 | -48 | 6 | 36 |
| L Fusiform Gyrus | 2 | 5.70 | 5.38 | -34 | -56 | -8 |
| L SFG | 3 | 5.66 | 5.38 | -30 | -2 | 60 |
| R Fusiform Gyrus | 6 | 5.61 | 5.36 | 22 | -80 | -16 |
| L IFG | 10 | 5.60 | 5.38 | -40 | 30 | 22 |
| L Lingual Gyrus | 2 | 5.51 | 5.42 | -16 | -54 | 44 |
| R MFG | 2 | 5.37 | 5.30 | 32 | 42 | 34 |

L SOG/R SOG: Left/Right Superior Occipital Gyrus; L MOG/R MOG: Left/Right Middle Occipital Gyrus; L IOG: Left Inferior Occipital Gyrus; R MFG/L MFG: Right/Left Middle Frontal Gyrus; L SFG: Left Superior Frontal Gyrus; L IFG: Left Inferior Frontal Gyrus.

**Supplementary Table S8. Brain Activation in Response to ‘Negative Emotional Valence’ Pictures in the Control Group as Compared to the PIGU group (Control Group > PIGU Group)**

| **Control Group > PIGU Group**  **(‘Negative Emotional Valence’) ; at pFWE<0.05** | | | | | | |
| --- | --- | --- | --- | --- | --- | --- |
|  |  |  |  | **Talairach coordinates** | | |
| **Regions** | **Voxel** | **Peak t** | **Mean t** | **X** | **Y** | **Z** |
| L Calcarine | 81 | 9.69 | 6.67 | -4 | -94 | 6 |
| R Calcarine | 154 | 8.07 | 6.09 | 16 | -80 | 14 |
| L Cuneus | 4 | 6.03 | 5.58 | -4 | -82 | 14 |
| R Middle Frontal Gyrus | 1 | 5.82 | 5.48 | 38 | -86 | 14 |
| L Lingual Gyrus | 7 | 5.79 | 5.43 | -6 | -56 | 2 |
| L Fusiform | 5 | 5.76 | 5.42 | -36 | -78 | -18 |
| R Inferior Occipital Gyrus | 4 | 5.68 | 5.43 | 26 | -94 | -6 |
| R Cuneus | 1 | 5.49 | 4.49 | 14 | -98 | 8 |
| R Lingual Gyrus | 1 | 5.19 | 5.19 | 6 | -60 | 6 |

L: Left; R: Right

**Supplementary Table S9. Correlation between PSC and IGAT Scores when presented with ‘Negative Emotional Valence’ cues in the PIGU Group**

|  |  | **L**  **dlPFC** | **L mPFC** | **L OFC** | **L Prec** | **R dlPFC** | **R mPFC** | **R OFC** | **R Prec** | **R**  **ACC** |
| --- | --- | --- | --- | --- | --- | --- | --- | --- | --- | --- |
| **IGAT** | **Spearman’s rho** | -0.099 | -0.198 | -0.002 | -0.045 | -0.369 | **-0.777*** | -0.376 | -0.232 | -0.673 |
|  | **Sig. (2-tailed)** | 0.726 | 0.480 | 0.995 | 0.874 | 0.176 | **0.001** | 0.167 | 0.405 | 0.006 |
|  | **N** | 15 | 15 | 15 | 15 | 15 | 15 | 15 | 15 | 15 |

*Correlation is significant at p < 0.004 level (2-tailed); Bonferroni corrected for multiple comparison

R dlPFC/L dlPFC: Right/ Left Dorso-lateral Prefrontal Cortex; L mPFC/R mPFC: Left/Right Medial Prefrontal Cortex; L OFC/R OFC: Left/Right Orbito-Frontal Cortex; RPrec/LPrec: Right/ Left Precuneus

|  |  | **L dlPFC** | **L mPFC** | **L OFC** | **L Prec** | **R dlPFC** | **R mPFC** | **R OFC** | **R Prec** | **R ACC** |
| --- | --- | --- | --- | --- | --- | --- | --- | --- | --- | --- |
| **IGAT** | **Spearman’s rho** | 0.150 | 0.289 | 0.173 | 0.040 | 0.013 | 0.162 | 0.456 | -0.159 | 0.155 |
|  | **Sig. (2-tailed)** | 0.594 | 0.297 | 0.537 | 0.888 | 0.964 | 0.563 | 0.087 | 0.572 | 0.581 |
|  | **N** | 15 | 15 | 15 | 15 | 15 | 15 | 15 | 15 | 15 |

**Supplementary Table S10. Correlation between PSC and IGAT Scores when presented with ‘Negative Emotional Valence’ cues in the Healthy Control Group**

*Correlation is significant at p < 0.004 level (2-tailed); Bonferroni corrected for multiple comparisons

R dlPFC/L dlPFC: Right/ Left Dorso-lateral Prefrontal Cortex; L mPFC/R mPFC: Left/Right Medial Prefrontal Cortex; L OFC/R OFC: Left/Right Orbito-Frontal Cortex; RPrec/LPrec: Right/ Left Precuneus
